# Changes in β-Cell Function in Offspring of Type-2 Diabetic Patients, as per Fasting and Two-Hour Plasma Glucose Levels

**DOI:** 10.7759/cureus.15056

**Published:** 2021-05-16

**Authors:** Edavan Pulikkanath Praveen, Sunil Chouhan, Jayaprakash Sahoo, Rajesh Khadgawat, Madan Lal Khurana, Nandita Gupta, Sada Nand Dwivedi, Bindu Kulshreshtha

**Affiliations:** 1 Biochemistry, Sindhudurg Shikshan Prasarak Mandal (SSPM) Medical College and Lifetime Hospital, Sindhudurg, IND; 2 Physiology, All India Institute of Medical Sciences (AIIMS), Bhopal, IND; 3 Endocrinology, Jawaharlal Institute of Postgraduate Medical Education & Research (JIPMER), Puducherry, IND; 4 Endocrinology, All India Institute of Medical Sciences (AIIMS), New Delhi, IND; 5 Biostatistics, All India Institute of Medical Sciences (AIIMS), New Delhi, IND; 6 Endocrinology, Atal Bihari Vajpayee Institute of Medical Sciences (ABVIMS), New Delhi, IND

**Keywords:** two-hour glucose categories, fasting glucose categories, offspring of subjects with t2dm, β-cell compensation, continuum, insulin sensitivity, first-phase insulin secretion, β-cell secretion

## Abstract

Background

The changes in β-cell function in high-risk populations who are apparently in the normal glucose tolerant stage are still under investigation for designing earlier prevention strategies. This study analyzes changes in β-cell function and insulin sensitivity across fasting and two-hour glucose categories spanning normal glucose tolerance (NGT) to impaired glucose tolerance (IGT), in offspring of subjects with type-2 diabetes mellitus (T2DM) compared to the controls without a known family history of T2DM.

Methods

Offspring of T2DM patients (cases) and individuals without a family history of T2DM (controls) were the subjects for this cross-sectional study. All participants underwent a 75 g oral glucose tolerance test and blood samples were collected for plasma glucose, insulin, C-peptide and proinsulin, at zero, 30, 60, and 120 minutes.

Results

A total of 358 cases (age 23.0 ± 10.8 years, 54% males) and 287 controls (age 28.4 ± 8.10 years, 65% males) were the subjects of this study. Cases and controls were divided into subgroups based on fasting and two-hour glucose categories spanning NGT to IGT. Compared to the reference category of controls (< 80 mg/dL for fasting glucose and < 84 mg/dL for two-hour glucose), cases with IGT had ~60% decline in both β-cell compensation (as measured as disposition index {0-120}) and insulin sensitivity (as measured as whole-body insulin sensitivity index {0-120}); adjusted for age, gender, and body mass index. From lower to higher fasting and two-hour glucose categories, there was a continuous and significant decline in β-cell compensation in both cases and controls. Significant reduction in first-phase insulin secretion, as measured as insulinogenic (0-30) index, was only observed among two-hour glucose categories, not among the fasting glucose categories. In the transition from late NGT cases to IGT cases, there was a significant decline in β-cell compensation, first-phase insulin secretion (more prominent than a decline in overall β-cell secretion) and the changes in whole-body insulin sensitivity were not statistically significant.

Conclusions

The decline in β-cell compensation was continuous and significant in offspring of subjects with type-2 diabetes and controls without a known family history of diabetes from early normal glucose tolerant ranges to impaired glucose tolerant ranges. Compared to the strictest glucose controlled category of controls, approximately 60% decline was observed in β-cell compensation and insulin sensitivity, in impaired glucose tolerant offspring of subjects with type-2 diabetes mellitus.

## Introduction

Maintenance of normal glucose tolerance depends on a finely tuned balance between insulin sensitivity and β-cell function [1]. The concept that a feedback loop governs the interaction of the insulin-sensitive tissues and the β-cell, as well as the elucidation of the hyperbolic relationship between insulin secretion and insulin sensitivity, explains the elevated insulin response in insulin-resistant subjects and a lower response in insulin-sensitive subjects [2]. Consideration of this hyperbolic relationship has helped to recognize the critical role of β-cell dysfunction, in the development of impaired glucose tolerance and type-2 diabetes [3]. Assessment of several ethnic groups has shown a progressive reduction in β-cell function from normal to impaired glucose tolerance and subsequently to type-2 diabetes, accompanied by a decline in insulin sensitivity [1,4]. The progressive nature of β-cell function in type-2 diabetes mellitus (T2DM) was also established in landmark clinical studies [5]. The therapeutic or lifestyle interventions should address the underlying pathology and should be started early along the spectrum of glucose tolerance to prevent declining insulin sensitivity and β-cell failure [6].

Normal glucose tolerance is expressed over a wide range of glucose concentrations; postprandial glucose concentration from about 70 mg/dL to 139 mg/dL. In normal glucose-tolerant subjects, insulin sensitivity and insulin secretion varied over a large range. According to a study in obese youth by Yeckel et al., insulin secretion as measured by the insulinogenic index has a strong impact on postprandial glucose levels even within the normal range, and in all insulin sensitivity tertiles [7]. Studies have suggested that reduced β-cell function manifested as reduced insulin release is a prerequisite for the progression from normal glucose tolerance (NGT) to hyperglycemia [8]. Most cross-sectional studies regarding the changes in β-cell function as a continuum from NGT to impaired glucose tolerance (IGT) were performed in older subjects or obese populations and the reference category was also similar. The offspring of type-2 diabetic patients of Indian origin is a younger population, vulnerable to hyperglycemia [9]. This cross-sectional study aimed to analyze the magnitude of changes in β-cell compensation, insulin sensitivity, first-phase insulin secretion, and proinsulin levels during oral glucose tolerance test, across fasting and two-hour glucose levels spanning NGT to IGT, in a relatively younger Indian origin population of offspring of subjects with T2DM, compared with a stricter glucose controlled group with no known family history of T2DM.

## Materials and methods

The participants in this research were descendants of patients with T2DM and people without a known family history of T2DM. Our sampling frame was the “offspring of individuals with T2DM” study database [10]. The study protocol was approved by the ethics committee of the All India Institute of Medical Sciences (AIIMS), New Delhi.

Recruitment of cases

Patients who were undergoing treatment for T2DM in the endocrine clinic of AIIMS, New Delhi, were informed about this study and asked to invite their children and grandchildren to participate. Only children and grandchildren with ages ranging from five to 55 years were included. Those with diabetes mellitus, pregnancy, lactation, or presence of any chronic illness were excluded.

Recruitment of controls

In addition to requiring age to be between five years and 55 years, controls had to have a negative history of T2DM in parents, siblings, and grandparents. Students and members of residents' associations of different areas were informed about the study with the help of a medical social worker. The study details were explained during group discussions. The exclusion criteria were the same as those for the cases. 

Detailed family history was recorded for all participants, with emphasis on the history of T2DM in parents, siblings, and grandparents. For the oral glucose tolerance test (OGTT), subjects were advised to maintain their normal diet and abstain from alcohol for three days prior to the test. After 10 to 12 hours of overnight fast, the OGTT was performed using a 75 g (1.75 g/kg body weight in the case of children, up to a maximum dose of 75 g) oral glucose dose. Blood samples were collected at zero, 30, 60, and 120 minutes after oral glucose dosing, from which to determine plasma glucose, insulin, C‐peptide, and proinsulin measurements. Glucose tolerance was determined by American Diabetes Association 2003 criteria [11].

Analytical measurements

Plasma glucose was measured by the glucose oxidase method on a Labmate-20 analyzer (Trivitron Diagnostics, Chennai, India). Plasma insulin and C-peptide were measured by electro-chemiluminescence assay by Elecsys 2010 (Roche Diagnostics, Indianapolis, USA). The insulin assay uses monoclonal antibodies against insulin and has 0.05% cross-reactivity with human proinsulin and its split forms. The intra-assay coefficient of variation (CV) for insulin assay was 5.1% and inter-assay CV was 5.7%. For C-peptide, intra-assay CV was 3.8% and inter-assay CV was 3.9%. Plasma proinsulin was measured by a radioimmunoassay kit (Catalog no. HPI-15K, Millipore Corporation, Billerica, MA). This assay cross-reacts neither with human insulin (<0.1%) nor with C-peptide (0.1%). It has 100% specificity for intact human proinsulin and 95% with des-31, 32 human proinsulin. Intra and inter-assay CV of proinsulin assay were 5.9% and 6.9%, respectively. 

The area under the curve (AUC) was calculated according to Tai’s formula. Area under the curve (AUC) = 15 [(x1+x2)+(x2+x3)+2(x3+x4)], where x1, x2, x3, and x4 were respective values on time points zero, 30, 60, and two-hours during OGTT [12]. Whole-body insulin sensitivity was measured as whole-body insulin sensitivity index (WBISI) as described by Matsuda et al [13]. Homeostasis model assessment-insulin resistance (HOMA-IR) was measured as proposed by Matthews et al. [14]. AUC of C-peptide (0-120) was divided by AUC of glucose (0-120) to determine insulinogenic index (0-120) [IGI 120], a measure of β-cell secretion, as described by Stadler et al. [15]. Disposition index (0-120) [DI 120] was calculated as the product of IGI 120 and WBISI, making a variation from the formula described by Retnakaran et al., [16] where plasma insulin was used in the place of C-peptide for the measurement of IGI 120. Insulinogenic index (0-30) (insulin in pmol/l {30-0 minute})/(glucose in mmol/l {30-0 minute}) was calculated as a measure of first-phase insulin secretion [17].

Statistical analysis

Statistical analysis was done using Statistical Package for the Social Sciences (SPSS) version 15 software (Chicago, IL: SPSS Inc.). For comparison of parameters between two groups, Student's unpaired t-test was used. A chi-square test was used to compare categorical variables. The quantitative continuous data were expressed as mean ± SD or mean ± SE values. Skewed data were normalized by applying log transformation for insulin, C-peptide, proinsulin, HOMA-IR, WBISI, IGI-120, IGI-30, and DI-120. The general linear model was used for comparing different groups after adjusting for confounding variables age, gender, and BMI. Post hoc comparison was done by Bonferroni's method. Tests were considered significant at p < 0.05.

## Results

A total of 645 subjects were participated in the study and underwent OGTT. Out of 645 subjects, 358 were with a family history of T2DM (cases) and 287 without a known family history of T2DM (controls).

There were 358 cases, among them, 301 were of normal glucose tolerance and 47 were having impaired glucose tolerance/impaired fasting glucose (IGT/IFG) and 10 subjects were newly diagnosed with diabetes. There were 287 cases, among them, 259 were normal glucose tolerant and 28 were having IGT/IFG.

The mean age of cases was 23.0 ± 10.8 (mean ± SD) years and that of controls was 28.4 ± 8.10 years (significantly higher age in controls, p < 0.001). Out of 358 cases, 194 (54%) were males and out of 287 controls, 187 (65%) were males (significantly higher percentage of males in controls, p = 0.006). There were 128 (35.6%) cases who had a mother with T2DM, 119 (33%) had a father with T2DM, 37 (10%) had both parents with T2DM and 74 (20.7%) of cases had one or more grandparents with T2DM (their parents were non-diabetic). Mean BMI of cases was higher 23.5 ± 6.0 (mean ± SD) kg/m^2^ (range 11.5-47.6) compared to 22.2 ± 3.9 kg/m^2^ of controls, (range 12-41.5; p = 0.001). 

Cases (n = 348) were divided into four groups according to fasting glucose (FG) and analyzed (10 newly diagnosed diabetic cases were excluded from this analysis due to limited numbers) (Table 1).

**Table 1:** Insulin sensitivity and β-cell function of cases (n = 348) according to fasting glucose, after adjusting for age, gender, and BMI. FG: fasting glucose; AUC: area under the curve; HOMA-IR: homeostasis model assessment-insulin resistance; WBISI: whole-body insulin sensitivity index; IGI: insulinogenic index

Parameters	FG ≤80 mg/dL n=81 mean ± SE	FG 81-90 mg/dL n=158 mean ± SE	FG 91-99 mg/dL n=81 mean ± SE	FG 100-126 mg/dL n=28 mean ± SE	p-Value
AUC proinsulin (nmolL^-^per 120 minute)	4.76 ± 0.24	4.89 ± 0.2	5.3 ± 0.3	6.8 ± 0.4	0.001
AUC proinsulin /AUC C-peptide	0.019 ± 0.001	0.019 ± 0.001	0.019 ± 0.001	0.021 ± 0.002	0.541
HOMA-IR (μU/mL, mmol/L)	1.7 ± 0.24	2.6 ± 0.17	3.0 ± 0.2	4.7 ± 0.4	< 0.001
WBISI (μU/mL, mg/dL)	8.85 ± 0.5	6.1 ± 0.4	4.9 ± 0.5	3.7 ± 0.9	< 0.001
IGI 120 (nmol/mmol)	0.40 ± 0.014	0.37 ± 0.009	0.38 ± 0.013	0.36 ± 0.023	0.825
IGI 30 (pmol/mmol)	244 ± 32	285 ± 22	310 ± 31	218 ± 54	0.407
Disposition index (0-120) (nmol/mmol, μU/mL, mg/dL)	2.7 ± 0.09	2.0 ± 0.06	1.6 ± 0.09	1.2 ± 0.16	< 0.001

On post hoc analysis, after adjusting for age, gender and BMI, compared to ≤ 80 categories of cases, there were significantly lower WBISI and disposition index (DI) 120 in 81-90, 91-99, and 100-126 mg/dl fasting glucose category of cases. WBISI and DI 120 were also significantly lower in 100-126 mg/dL compared to 81-90 and 91-99 mg/dL fasting categories. Insulinogenic indices were not significantly different. The area under the curve of proinsulin was significantly increased in IGT ranges compared to early NGT ranges. 

**Figure 1:**
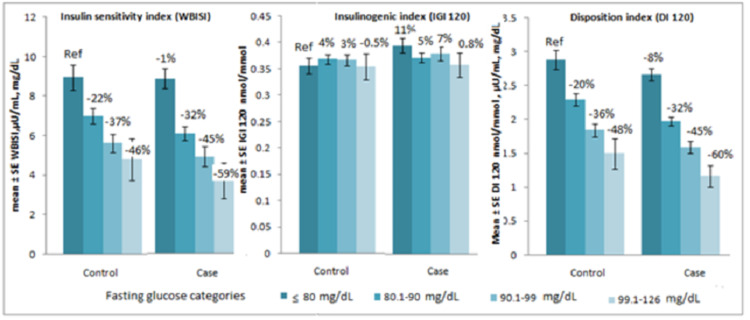
WBISI, IGI 120, and DI 120 according to fasting glucose categories in cases and controls, after adjusting for age, gender, and BMI. WBISI: whole-body insulin sensitivity index; IGI: insulinogenic index; DI: disposition index

Controls were also divided into four groups based on FG levels as done for cases. Among controls, there were 49 subjects in ≤ 80, 128 in 81-90, 91 in 91-99 and 19 subjects in 100-126 mg/dL FG categories. FG ≤ 80 mg/dL was taken as a reference category. The percentage of reduction in WBISI, insulinogenic index (IGI 120) and disposition index (DI 120) in FG 81- 90 and 91-99 and 100-126 mg/dL categories in cases (n = 348) and controls (n = 287) were calculated (Figure 1). The analysis omitted 10 cases of newly diagnosed with type 2 diabetes due to limited numbers. 

There was a gradual decline in insulin sensitivity and disposition index in both cases and controls as per the FG categories. In each category, cases have lower insulin sensitivity compared to controls.

Cases (n = 348) were divided into four groups according to two-hour glucose levels and were analyzed (10 diabetic cases were excluded from this analysis due to small numbers) (Table 2).

**Table 2:** Insulin sensitivity and β-cell function of cases (n = 348) according to two-hour glucose categories, after adjusting for age, gender, and BMI. AUC: area under the curve; HOMA-IR: homeostasis model assessment-insulin resistance; WBISI: whole-body insulin sensitivity index; IGI: insulinogenic index

Parameters	2-hour glucose < 100 mg/dL n = 201 mean ± SE	2-hour glucose 100-119 mg/dL n = 92 mean ± SE	2-hour glucose 120-139 mg/dL n = 30 mean ± SE	2-hour glucose >139 mg/dL n = 25 mean ± SE	p-Value
AUC proinsulin (nmolL^-1^ per 120 minute)	4.65 ± 0.15	5.5 ± 0.23	6.1 ± 0.4	6.2 ± 0.4	0.001
AUC proinsulin/AUC C-peptide	0.019 ± 0.001	0.019 ± 0.001	0.021 ± 0.001	0.020 ± 0.002	0.430
HOMA-IR (μU/mL,mmol/L)	2.44 ± 0.16	2.77 ± 0.23	3.03 ± 0.41	3.48 ± 0.45	< 0.001
WBISI (μU/mL, mg/dL)	7.3 ± 0.3	4.8 ± 0.5	5.1 ± 0.8	4.2 ± 0.9	< 0.001
IGI 120 (nmol/mmol)	0.383 ± 0.008	0.396 ± 0.012	0.37 ± 0.022	0.297 ± 0.023	0.005
IGI 30 (pmol/mmol)	325 ± 20	236 ± 28	249.5 ± 49	100.3 ± 53	< 0.001
Disposition index (0-120) (nmol/mmol, μU/mL, mg/dL)	2.3 ± 0.06	1.66 ± 0.08	1.52 ± 0.15	1.1 ± 0.16	< 0.001

On post hoc analysis, there were significantly higher AUC of proinsulin levels in 100-119, 120-139, >139 mg/dL categories compared to < 100 mg/dL, two-hour glucose category. HOMA-IR was significantly higher in 100-119 and > 139 categories compared to <100 mg/dL category. WBISI of 100-119, 120-139 and >139 mg/dL categories were significantly lower compared to < 100 mg/dL category. Between 100-119, 120-139, and > 139 mg/dL categories, WBISI was not significantly different. IGI (0-120) was significantly lower in the > 139 mg/dL category compared to 100-119 and < 100 mg/dL and other comparisons were not significantly different. Compared to < 100 mg/dL two-hour glucose category, disposition index was significantly lower in 100-119, 120-139 and > 139 mg/dl two-hour glucose categories. Disposition index of > 139 mg/dL two-hour category was significantly lower compared to other lower glucose categories too. 

Cases and controls were divided into smaller categories for analyzing the trends and magnitude of changes in β-cell compensation and insulin sensitivity according to two-hour glucose levels. The subjects were divided into eight categories according to glucose at two hours, spanning from NGT to IGT/DM. In cases, there were 71 subjects in < 84 mg/dL (two-hour glucose ), 98 in 84-95, 77 in 96-108, 35 in 109-115, 27 in 116-125, 17 in 126-139, 16 in 140-155, and 17 subjects in > 155 mg/dL category. In controls, there were 73 subjects in < 84 mg/dL (two-hour glucose category), 55 in 84-95, 68 in 96-108, 24 in 109-115, 40 in 116-125, 16 in 126-139, and nine subjects in 140-155 mg/dL two-hour glucose category.

**Figure 2:**
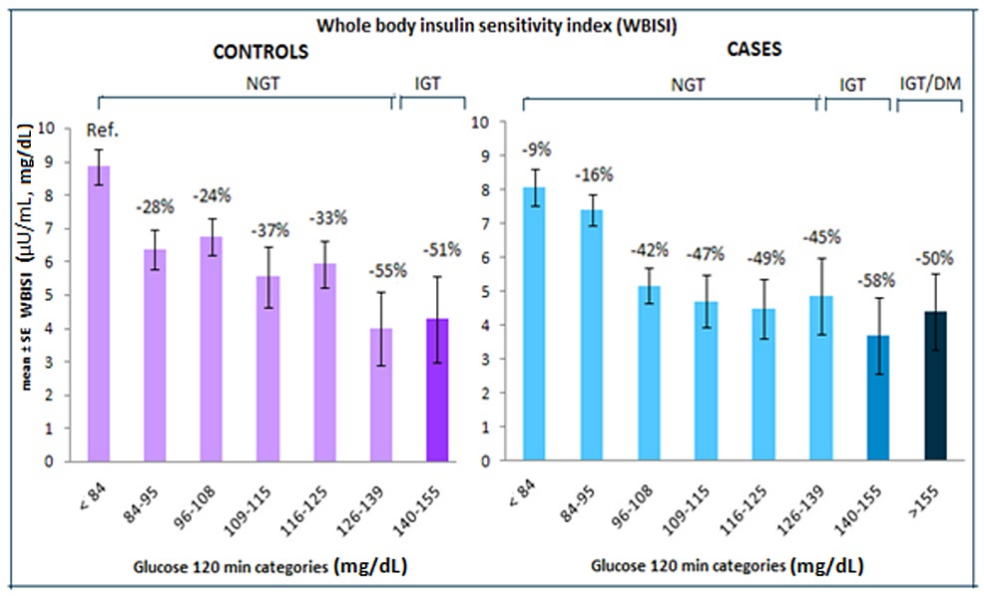
Change in WBISI in controls and cases across two-hour glucose categories, data were adjusted for age, gender, and BMI. WBISI: whole-body insulin sensitivity index; NGT: normal glucose tolerance; IGT: impaired glucose tolerance; DM: diabetes mellitus

Less than 84 mg/dL two-hour glucose category of controls was taken as the reference category. The percentage of reduction in WBISI in different two-hour glucose categories from the reference group was given in Figure 2.

Both in cases and controls, there was a decline in insulin sensitivity across two-hour glucose categories however it was not a steady decline in both cases and controls. Overall lower insulin sensitivity was observed in cases compared to controls in the middle NGT ranges. From 126-139 mg/dL (two-hour glucose) onwards insulin sensitivity seems to be similar in both cases and controls. With a decline of insulin sensitivity of 47% in the 109-115 category compared to the reference category (p = 0.02), there was no further significant change in late NGT and IGT/new DM categories of cases. 

**Figure 3:**
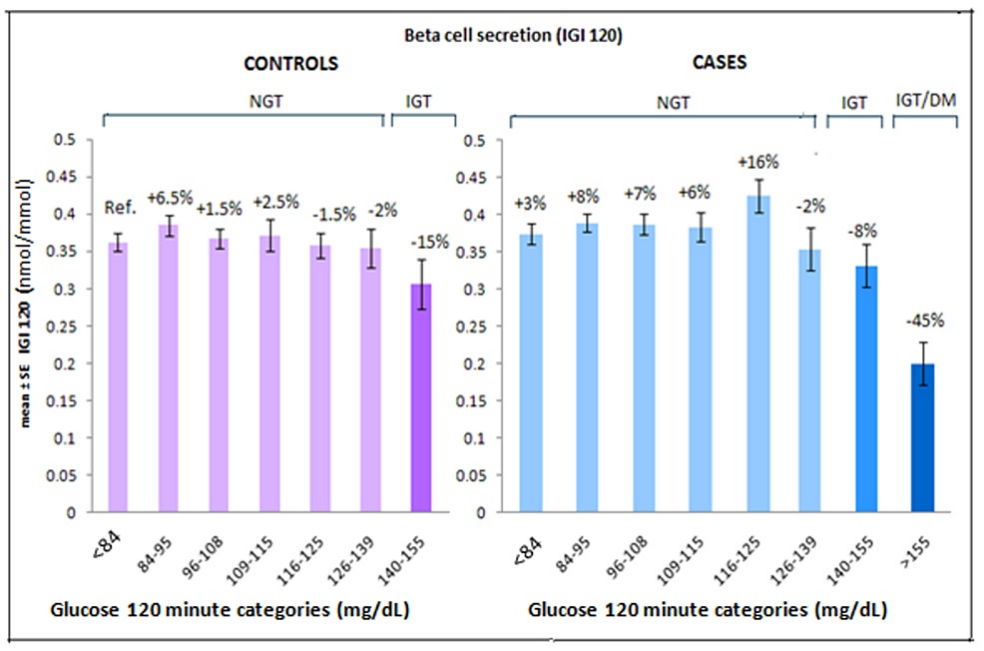
Change in β-cell secretion (IGI 120) in controls and cases across two-hour glucose categories, data were adjusted for age, gender, and BMI. IGI: insulinogenic index; NGT: normal glucose tolerance; IGT: impaired glucose tolerance; DM: diabetes mellitus

Compared to the two-hour reference category (< 84 mg/dL of controls), there was a trend of increase in β-cell secretion both in cases and controls in early NGT ranges, a trend of decline was observed from late NGT ranges 126-139 mg/dL). A further decline was observed in IGT/new DM range (8-45%) (Figure 3).

**Figure 4:**
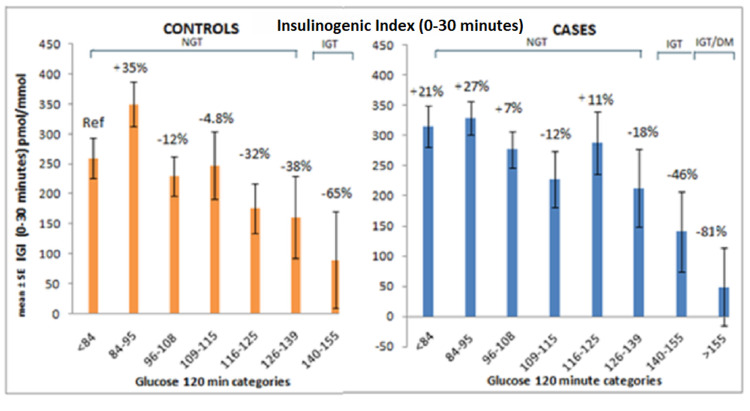
Changes in Insulinogenic index (0-30) in controls and cases across two-hour glucose categories, data were adjusted for age, gender, and BMI. IGI: insulinogenic index; NGT: normal glucose tolerance; IGT: impaired glucose tolerance; DM: diabetes mellitus

There was a decline in first-phase insulin secretion as measured by IGI 30 of cases and controls compared to the reference category. In cases, the decline in IGI-30 was observed from 126-139 mg/dL two-hour glucose category and this seems to be in parallel with changes in the insulinogenic index (0-120), however, the decline was sharper (Figure 4).

**Figure 5:**
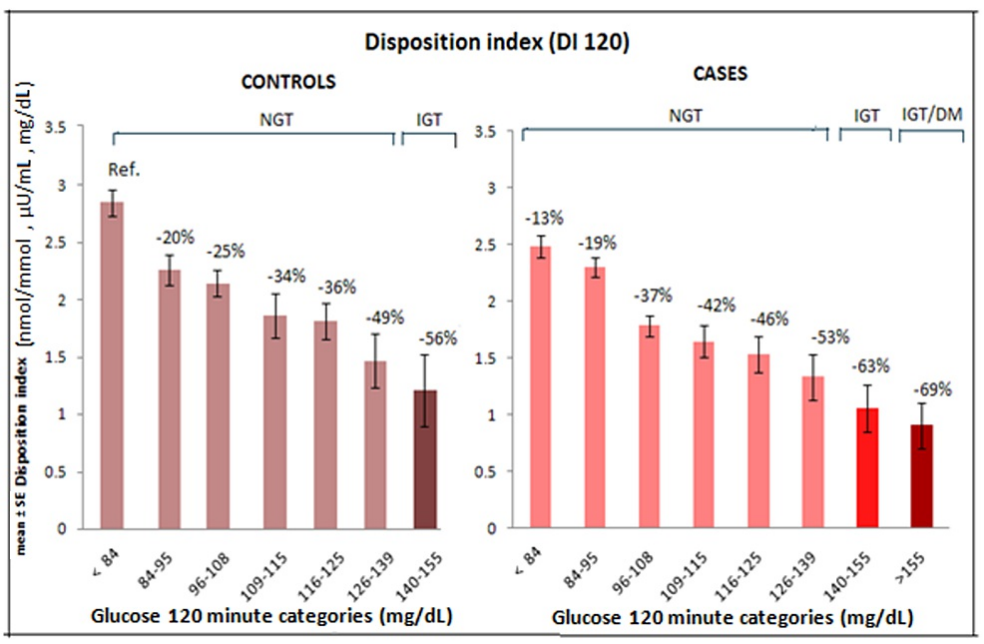
Change in β-cell compensation (DI 120) in controls and cases across 120-minute glucose categories, data were adjusted for age, gender, and BMI. NGT: normal glucose tolerance; IGT: impaired glucose tolerance; DM: diabetes mellitus

There was a continuous and significant decline in β-cell compensation as measured by DI 120 in both controls and cases (p < 0.001; Figure 5).

In both cases and controls, there was a continuous decline in DI120 as two-hour glucose levels increased. Approximately 50% reduction was observed in 126-139 category and a reduction of approximately 60% was observed in IGT two-hour ranges in cases compared to the reference two-hour category. 

For studying the transition from normal to impaired glucose tolerance, normal glucose tolerant cases with two-hour glucose 116-139 mg/dL group were compared with IGT/IFG cases (Table 3). NGT cases with two-hour glucose 116-125 and 126-139 mg/dL categories were combined into one group and compared with IGT/IFG group.

**Table 3:** Comparison of Insulin sensitivity and β-cell function between cases with two-hour glucose 116-139 mg/dL and IGT/IFG cases. Data are adjusted for age, gender, and BMI. AUC: area under the curve; HOMA-IR: homeostasis model assessment-insulin resistance; WBISI: whole-body insulin sensitivity index; IGI: insulinogenic index; NGT: normal glucose tolerance; IFG:

	NGT cases with 2-hour glucose (116-139) n = 33 mean ± SE	IGT/IFG cases n = 47 mean ± SE	p-Value
Plasma insulin 0 minutes (µU/mL)	14.9 ±1.8	16.8 ±1.5	0.189
AUC glucose (mmolL^-1^ per 120 minutes)	863 ± 23	995 ± 19	< 0.001
AUC proinsulin (nmolL^-1^ per 120 minutes	6.38 ± 0.37	6.39 ± 0.29	0.765
AUC proinsulin/AUC C-peptide	0.018 ± 0.001	0.018 ± 0.001	0.851
HOMA-IR (μU/mL, mmol/L)	3.3 ± 0.4	4.1 ± 0.37	0.051
WBISI (μU/mL, mg/dL)	3.7 ± 0.28	2.9 ± 0.23	0.116
IGI 120 (nmol/mmol)	0.45 ± 0.02	0.37 ± 0.02	0.013
IGI 30 (pmol/mmol)	276.6 ± 37.6	183 ± 30.7	0.004
Disposition index (0-120) (nmol/mmol, μU/mL, mg/dL)	1.4 ± 0.07	1.0 ± 0.06	< 0.001

Insulin resistance as measured by HOMA-IR was higher in IGT/IFG group, had shown borderline significance (p = 0.051), however, WBISI was not significantly different between late NGT and IGT/IFG. AUC of proinsulin and AUC proinsulin to AUC C-peptide ratio was also not significantly different. A significant reduction was observed in IGI 30 (33% reduction) and IGI120 (18% reduction) and DI 120 (28% reduction) in IGT, compared to the late NGT group.

## Discussion

Type-2 diabetes mellitus is a global health concern. In the developed nations, it affects predominantly middle-aged and older populations; while in Asian countries like India and communities vulnerable to malnutrition, it affects the younger population [9]. The challenge to attain and maintain normoglycemia is compounded by the progressive nature of β-cell failure, which starts many years before the diagnosis of diabetes from the prediabetic stage or probably even before [3,18]. Subjects with a family history of T2DM are an ideal group to study the earliest abnormalities, as they are at higher risk for diabetes and abnormalities could be starting at a younger age [15,19]. In the present study, the mean age of subjects was in their twenties, reducing the confounding effects due to age. Whole-body insulin sensitivity in the present study was measured as WBISI as described by Matsuda and DeFronzo [13], which was shown to be having an excellent correlation with euglycemic hyperinsulinemic clamp technique by large scale studies [20]. The calculated disposition index by OGTT highlighted the inability of β-cell to compensate for declining insulin sensitivity [16]. In prospective studies, the disposition index declined well before glucose levels rise into the diabetic range, was mentioned as an early marker for inadequate β-cell compensation or β-cell dysfunction [21]. 

Metabolic Syndrome in Men (METSIM) study of the Finnish population was a large-scale study to evaluate the insulin sensitivity and insulin secretion over the entire range of fasting and two-hour plasma glucose, including diabetes [20]. The subjects were older and had higher BMI (mean age: 57 years, mean BMI: 27 kg/m^2^). They used WBISI as a measure of insulin sensitivity, IGI 30 for early-phase insulin release, AUC insulin/AUC glucose as a marker for total insulin release, and disposition index (DI120 and DI30) as a β-cell response to insulin sensitivity. There was a ~20% decline WBISI and ~24% decline in DI 120 in the 90-97 mg/dL FG range compared to the reference range of < 90 mg/dL [20]. In the present study, subjects with a family history of T2DM (cases) were analyzed separately and compared with the reference group selected from controls. There was around 45% decline in insulin sensitivity and β-cell compensation in 90.1-99 mg/dL FG category of cases compared to < 80 mg/dL reference category. In the IGT ranges, the reduction in insulin sensitivity and β-cell compensation was ~60%. Compared to the 80-90 mg/dL FG group, cases in the 90-99 group has ~23% decline in insulin sensitivity and disposition index and were comparable to the METSIM study. A study by Dagogo-Jack et al., divided subjects having normal fasting glucose into two groups, low normal fasting (low normal fasting glucose {NFG} < 90 mg/dL) and high-normal fasting (high NFG 90-99 mg/dL, n = 13) and compared with impaired FG and combined IFT-IGT [22]. Compared to the low-normal FG group, the disposition index decreased by 33% in the high normal FG group, 46% in i-IFG subjects, and 50% in those with combined IFG-IGT. Three-fold variability in insulin sensitivity was reported in the 70-125 mg/dL FG range [22]. In the present study, after adjusting for age, gender, and BMI, the variation in the mean insulin sensitivity was around 2.5 folds in the 70-125 mg/dL FG range. We observed lower insulin sensitivity and β-cell compensation in FG categories in cases compared to controls. This was similar to a study by Ehrmann et al., conducted in polycystic ovary syndrome (PCOS) subjects. PCOS with a family history of T2DM has shown a lower β-cell compensation for the degree of insulin resistance at all FG categories studied within the NGT range, compared to PCOS subjects without a family history of T2DM and women with no history of PCOS [23]. Proinsulin levels showed a progressive and significant increase from early NGT to IGT ranges in the current study. Significant hypersecretion of proinsulin with respect to the secretion of C-peptide levels during OGTT was not observed in the late NGT ranges, even though there was a trend of higher proinsulin to C-peptide ratio in IGT ranges. Studies have observed higher proinsulin to insulin ratio in diabetics, which could be because of inappropriate proinsulin processing due to various reasons like increased secretory demand on β-cell by chronic hyperglycemia or due to primary defect in the synthesis or secretion machinery [24]. As per our observations, unlike established diabetes, abnormal proinsulin processing may not be a feature of late NGT stages and the trend of relative hypersecretion of proinsulin may be starting from the IGT state.

San Antonio Metabolism (SAM) study had observed a greater than 50% decline in the plasma insulin response to a glucose challenge in NGT individuals with two-hour plasma glucose between 110 to 140 mg/dL. IGT subjects with two-hour plasma glucose between 140 mg/dL and 200 mg/dL manifested a severely impaired plasma insulin response. The mean age of subjects was above 35 years in different categories [25]. In the METSIM study, the whole-body insulin sensitivity index (WBISI) reduced 37% in the NGT range and 44% reduction in the IGT range, as a function of two-hour glucose levels. In the IGT and DM ranges, WBISI was almost similar. They observed a 30% decline in disposition index (DI 120) in the late normal range and further decreased to 48% in the IGT range (< 90 mg% categories was taken as reference) [20]. Burns et al. studied overweight youth, showed >30% decline in β-cell function relative to insulin sensitivity in late NGT ranges and ~40% in IGT ranges, compared to < 120 mg/dL two-hour group [26]. In the current study, there was a 45% decline in insulin sensitivity in the late NGT ranges and a 50% to 60% decline in the IGT ranges compared to the two-hour glucose reference category of < 84 mg/dL of controls. There was a decline of 53% in the disposition index in the late NGT and ~63% decline in the disposition index in the IGT range. The higher reduction in insulin sensitivity and β-cell compensation in the current study compared to the above-mentioned studies could be due to the stricter glucose-controlled reference category selection. In the cases, insulin sensitivity was similar in the late NGT ranges and IGT/DM giving similar kind of observations to that of the METSIM study [20]. The decline in overall β-cell secretion and first-phase insulin secretion in the background of already reduced insulin sensitivity (more prominent in cases, 40-50% reduction in WBISI) could be the reason for reduced β-cell compensation and subsequent elevation of blood glucose levels in the late NGT and IGT/DM subjects. This was also evident when we compared the late NGT group and IGT/IFG after adjusting for age, gender, and BMI. Whole-body insulin sensitivity was not significantly different between the groups, however, there was a significant reduction in overall β-cell secretion (p = 0.013), first-phase insulin secretion (p = 0.004), and β-cell compensation (p < 0.001) in the IGT ranges. 

Overall β-cell secretion (IGI 120) and first-phase insulin secretion (IGI 0-30) were not significantly different in case subjects across the FG category, however across two-hour glucose levels, there was a significant reduction in both IGI 120 (p = 0.005) and IGI 30 (p < 0.001). Compared to the increase in fasting glucose levels, an increase in glucose at two-hour may better reflect the reduction in overall β-cell secretion and first-phase insulin secretion. The magnitude of decline was more in first-phase insulin secretion than in overall β-cell secretion (46% vs. 8%) in the IGT range, compared to the reference two-hour category of < 84 mg/dL. This is in agreement with a large-scale study conducted by Aoyama-Sasabe et al., in the Japanese population, where they have observed, a reduction in early-phase insulin secretion as the most important factor responsible for elevation of two-hour glucose levels during OGTT in isolated IGT subjects [27]. Restoration of first-phase insulin secretion by dietary and therapeutic means has been proposed as a method not only for the prevention of diabetes but also for the prevention of cardiovascular events [28].

Controls for this study were subjects without a family history of T2DM. However, family members of these subjects were not tested for T2DM. Some may have undiagnosed T2DM as routine periodic health check-up is not common in this region. Cases and controls were not matched for gender and age (mean age 23 years vs. 28 years, higher in controls, p < 0.001), which was a big limitation of the study. To reduce the confounding effects, the analysis was matched for age, gender, and BMI. Being a cross-sectional study, a cause-effect relationship could not be deduced from the present study.

## Conclusions

In conclusion, we had observed a continuous and significant decline in β-cell compensation starting from normal glucose tolerance to impaired glucose tolerance in both offspring of individuals with type-2 diabetes (cases) and controls. In the cases, the reduction in insulin sensitivity was not continuous from NGT to IGT ranges and was not significantly different between late NGT and IGT levels. The decline in first-phase insulin secretion was more pronounced than overall β-cell secretion in the IGT levels, compared to the late NGT state in cases. Even though the proinsulin levels during OGTT were increased from NGT to IGT ranges, a significant difference in relative hyperproinsulinemia with respect to the C-peptide levels was not observed between late NGT and IGT levels. Compared to a stricter glucose control group without a known family history of type-2 diabetes mellitus, based on both fasting and two-hour plasma glucose levels, approximately 60% decline was observed in β-cell compensation and insulin sensitivity, in offspring of T2DM subjects in the impaired glucose tolerant state.
